# Inpatient dialectical behavior therapy combined with trauma-focused therapy for PTSD and borderline personality disorder symptoms: study design of the naturalistic trauma therapy study

**DOI:** 10.3389/fpsyt.2025.1538267

**Published:** 2025-02-05

**Authors:** Annemieke C. Kamstra, Sybolt O. de Vries, Maarten F. Brilman, Petty Vasilev, Manna A. Alma, Antoinette D. I. van Asselt, Mia De Wolf, Robert A. Schoevers, Frederike Jörg

**Affiliations:** ^1^ Research Department, GGZ Friesland, Leeuwarden, Netherlands; ^2^ Department of Psychiatry, Interdisciplinary Center Psychopathology and Emotion Regulation, University Medical Center Groningen, University of Groningen, Groningen, Netherlands; ^3^ Department of Health Sciences, University Medical Center Groningen, University of Groningen, Groningen, Netherlands; ^4^ Department of Epidemiology, University Medical Center Groningen, University of Groningen, Groningen, Netherlands

**Keywords:** DBT-PTSD, study protocol, dialectical behavior therapy, post-traumatic stress disorder, parasuicidal behavior, borderline personality disorder, cost-effectiveness

## Abstract

**Introduction:**

Childhood traumatization can result in physical and mental health problems in adulthood, such as post-traumatic stress disorder (PTSD), which negatively influences quality of life and social functioning. Although evidence based trauma treatments benefit clients with PTSD after childhood abuse and comorbid personality disorders, they are less effective than in clients who were traumatized in adulthood, and drop-out is substantial. The current study aims to assess the effects of inpatient dialectical behavior therapy combined with prolonged exposure (DBT-PTSD) on severity of PTSD, dissociation, parasuicidal behavior and borderline personality disorder (BPD) in clients with severe PTSD and comorbid psychiatric disorders. Secondary outcomes are social functioning, quality of life, borderline and cluster C personality disorder symptoms as treatment predictors, treatment trajectories, clients’ experiences and health economic consequences.

**Methods:**

The naturalistic, longitudinal Trauma Therapy Study is conducted from January 2019 until May 2025 in a mental healthcare center in the Netherlands. Clients with severe PTSD and comorbid conditions who are referred to inpatient DBT-PTSD are included into the study. Based on power analyses a total sample size of N=56 is needed. Measurements take place before the waiting list period, at pre- and posttreatment and at six- and twelve-months follow-up. Clients fill in a daily DBT-PTSD diary, which gives insight into individual symptom trajectories.

**Results:**

Statistical analyses include two-sided paired samples t-tests, linear mixed model analyses and cost-effectiveness analyses. Qualitative interviews are conducted within two years posttreatment and analyzed using a phenomenological approach. We correct for chance capitalization by using a conservative α-level of.01. Multiple imputation is used to handle missing data.

**Discussion:**

Research on the effects of integrated treatment programs for clients with severe PTSD and co-morbid conditions is scarce. This study extends current knowledge on the effects of inpatient DBT-PTSD on PTSD and BPD symptoms, clients’ social functioning and quality of life. In addition, it provides insight into individual symptom trajectories and experiences, inspiring future treatment improvements for clients with severe psychopathology.

**Trial registration:**

Medical Ethical Committee approval (NL669060018, RTPO1044/01.10.2018). Preregistration: Dutch registration database Centrale Commissie Mensgebonden Onderzoek and International Clinical Trials Registry Platform (NL-OMON46167/01.10.2018/https://trialsearch.who.int/Trial2.aspx?TrialID=NL-OMON46167).

## 1 Introduction

Adverse childhood experiences, like physical, sexual or emotional abuse can lead to physical and mental health problems in adulthood (1), including post-traumatic stress disorder (PTSD) or complex PTSD (see **Box 1**). PTSD symptoms, like intrusions, avoidance, negative trauma-related cognitions and mood and hyperarousal (4) have a great negative impact on quality of life (5) and social functioning (6, 7). PTSD has a life-time prevalence of 3.9% (8) and often co-occurs with mental health problems, such as depression, anxiety, dissociation, substance use disorders, eating disorders and borderline personality disorder (BPD) (9–13). Clients with histories of severe childhood trauma seem to experience more severe PTSD symptoms and dissociation (14, 15), have more trouble with emotion regulation (14) and report a poor quality of life (5). Also, a greater number of childhood trauma types is related to a poorer mental and physical quality of life (16).

Box 1 The International Classification of Diseases 11th Revision (ICD-11) differentiates between PTSD, complex PTSD and borderline personality disorder. Complex PTSD includes negative self-concept, relational problems and emotion regulation difficulties (2). Although the classifications complex PTSD and borderline personality disorder seem to overlap, most studies conclude that they are distinct classifications (3). The Diagnostic and Statistical Manual of Mental Disorders, Fifth Edition Text Revision (DSM-5-TR) classifies PTSD as a single condition (4). Since the mental healthcare system of the Netherlands uses the DSM-5-TR, in this study we define PTSD and BPD accordingly.

Although clients with PTSD after childhood abuse do benefit from evidence-based treatments (17, 18), there is room for improvement. For one, pooled drop-out rates vary between 16% and 41.5% (19, 20), appear to be greater in trauma-focused therapies and with childhood abuse-related PTSD (19, 21). Further, symptoms seem to improve to a lesser extent after interpersonal trauma (22), for clients with complex PTSD after childhood (sexual) abuse (23) and for clients with comorbid personality disorders (24). Also, emotion regulation and quality of life do not automatically improve after PTSD treatment, as shown in a systematic review and meta-analysis (18). For example, women with PTSD after childhood trauma reported very modest quality of life improvements that moved from ‘very low’ before Cognitive Processing Therapy to ‘low’ afterwards (5). Recently, more integrated and personalized interventions were suggested to improve treatment results (25, 26).

One such approach is the integration of Dialectical Behaviour Therapy (DBT) and Prolonged Exposure (PE). Several groups have studied a combination of PE and DBT, to make PTSD treatment accessible for clients with complex psychopathology, including high-risk behavior (27), and to address the needs of clients with chronic PTSD after childhood sexual abuse (28, 29). A systematic review and meta-analysis of inpatient and outpatient treatments that combine DBT and PE finds that PTSD and depressive symptoms decrease (30). Steil et al. (2011) developed inpatient DBT-PTSD for clients with severe PTSD after childhood sexual abuse. This treatment program of 12 weeks is based on DBT principles, includes PE as primary trauma-focused intervention and incorporates elements of Acceptance and Commitment Therapy and Compassion Focused Therapy (31). In a pilot (N=29) and RCT (N=74), with a Treatment As Usual (TAU) waiting list condition, women with and without comorbid BPD reported significant and clinically relevant improvements in PTSD after inpatient DBT-PTSD (28, 29). Drop-out was low in both RCT groups (DBT-PTSD: N=2, 5.5%; Control: N=3, 7.9%). Finally, in an observational study clients reported more improvement in PTSD symptoms, dissociation and disturbances in self-organization after inpatient DBT-PTSD than those treated with an inpatient TAU that included group interventions and trauma-focused treatment (32).

So far, these results of integrating DBT and PE are promising for clients with severe PTSD, comorbid BPD and a history of childhood sexual abuse. At the same time, studies on inpatient DBT-PTSD are still rather scarce. Replication of the results in various clinical settings and with a greater variation in PTSD client populations, regarding gender and a wider variety of trauma histories, are needed to inform treatment guidelines and clinical practice. Furthermore, little is known about predictors of treatment. In general, clients with PTSD and comorbid personality disorders seem to benefit somewhat less from PTSD treatments (24), but exploratory analyses showed that comorbid BPD was not predictive of DBT-PTSD treatment outcome (29). To improve our knowledge about client characteristics that may predict treatment outcome in DBT-PTSD, it is important that other personality disorders are also taken into account. In addition, obtaining a better insight into client perspectives and experiences with DBT-PTSD may also help improve this treatment. Finally, we are interested in the economic impact of DBT-PTSD, because the economic consequences of childhood trauma in terms of productivity loss and health care cost are high and increase with greater severity and complexity of the associated psychopathology (33–35).

The primary aim of this longitudinal, within-subjects study is to assess the effect of inpatient DBT-PTSD on PTSD severity in clients with severe PTSD and comorbid disorders. Secondary outcomes are dissociation, parasuicidal behavior, comorbid BPD symptoms, social functioning and quality of life. We hypothesize that PTSD, dissociation, parasuicidal behavior and BPD symptoms will decrease significantly after DBT-PTSD, in contrast to no significant changes after the waiting list period. Since DBT-PTSD offers training and therapy beyond trauma-focused treatment, we hypothesize improvements in quality of life and social as well. Especially at the six and twelve months follow-up measurements, as clients were able to pick up their daily lives after leaving the clinic. A more exploratory aim is to study whether BPD and cluster C personality disorder symptoms predict the effects of DBT-PTSD on PTSD symptoms. The fourth objective is to study the cost-effectiveness of inpatient DBT-PTSD. Finally, we intend to study clients’ experiences with the treatment program and, more exploratory, the individual differences in symptom trajectories.

## 2 Methods

### 2.1 Study design

**Figure 1:**
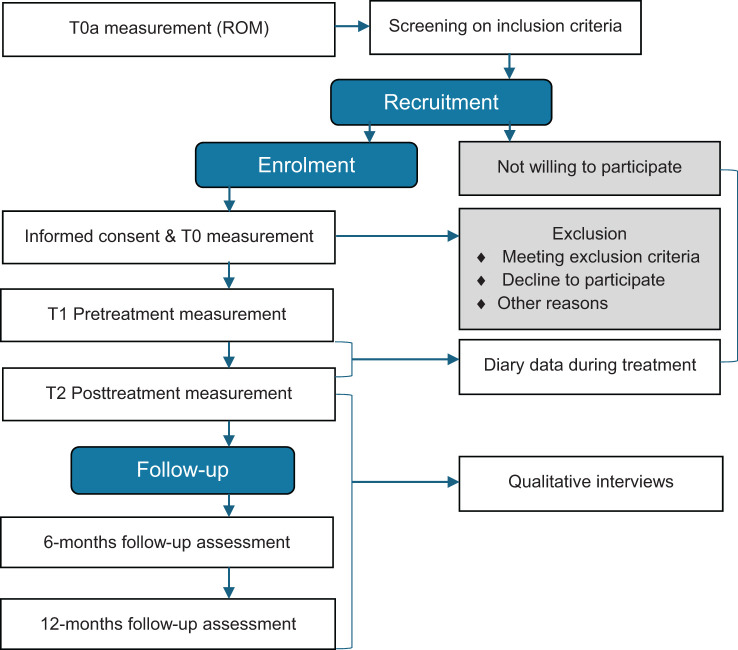
Flowchart of the Trauma Therapy Study procedure.

The Trauma Therapy Study (TTS) is a naturalistic, longitudinal study with a within-subjects design. It takes place at GGZ Friesland, a mental health institute in The Netherlands from January 2019 to May 2025. Clients referred to inpatient DBT-PTSD are assessed at the beginning of the waiting list period (T0), at pre (T1) and posttreatment (T2) and at six (T3) and twelve months (T4) follow-up (**Figure 1**). The waiting list period is estimated to be approximately 12 weeks, based on the year before the start of the study. It may vary in length depending on the number of referrals to inpatient DBT-PTSD and unforeseen circumstances. When the waiting list period exceeds the three months, the baseline measurements are repeated to create a three months period between baseline and start of the intervention. Pre to posttreatment differences are compared to the control period, the pre to post waiting list period. During this period clients could continue any form of TAU to treat PTSD, as this is the case in everyday clinical practice.

### 2.2 Procedure

Clients eligible for DBT-PTSD are first informed about the study during the commitment interview. The importance of truly committing to the treatment is then discussed and afterwards clients committed to DBT-PTSD are then placed on the waiting list. Those interested in study participation are sent detailed written information and receive a follow-up telephone call to provide further information. Clients are informed that study participation entails extra questionnaires and interviews, beside the standard diagnostic procedures and Routine Outcome Measurements (ROM).

During the baseline assessment (T0), informed consent is obtained by the research nurse. Interviews are performed by independent research assistants and clinical psychologists (in training). The interviewers are trained in the use of the instruments and regularly attend group intervision meetings to enhance reliability. Clients complete self-report questionnaires online. To improve study participation and retention clients can choose between being interviewed at the mental healthcare facility of via video call, This is particularly convenient for those living far away from the treatment center. We also approach clients enrolled in the TTS who finished DBT-PTSD in the previous two years and invite them for a qualitative interview about their experiences. These interviews are conducted by two clinical psychologists in training (AK and PV) who are unacquainted with these clients. Clients give an additional informed consent before starting this interview. Finally, we use the DBT-PTSD diary data from clients that started DBT-PTSD between 2019 (when the diary was digitalized) and December 2023 to study symptom trajectories. Clients fill in this daily diary as part of the treatment program.

### 2.3 Ethical statement

The Medical Ethical Committee (METc) granted ethical approval for the second version of this protocol (nr. NL669060018, RTPO1044, date: 1 October 2018) and the subsequent amendment of 2 September 2022, to include a qualitative study. The study is preregistered at the Dutch registration database Centrale Commissie Mensgebonden Onderzoek and the International Clinical Trials Registry Platform (NL-OMON46167) and at the Center for Open Science (osf.io/74be8, https://doi.org/10.17605/OSF.IO/W3BHZ).

Clients are informed that they can stop study participation at any moment. Adverse events are recorded and serious adverse events are reported to the sponsor and the MEtc. The sponsor will suspend the study if there is sufficient ground that continuation of the study will jeopardize participants’ health or safety. Anonymized ROM assessments of non-participants is used to test for selection bias, except when clients opted-out and wanted their data removed from the research database of the mental health institute. This applies to the anonymized use of diary data of both participants and non-participants as well. As a standard procedure at GGZ Friesland, a colleague of the research department who is not involved in the project oversees the progress of the study. We followed the SPIRIT writing guidelines (36) and added the SPIRIT checklist to the submission of this study protocol.

### 2.4 Participants

Clients aged 18–65 are eligible for inpatient DBT-PTSD if they meet the DSM-5 diagnostic criteria for PTSD and suffer from severe psychopathology. This includes various comorbid disorders. Trauma type is not leading in the indication process, but clients referred to the treatment center have often suffered from severe interpersonal traumas during childhood. Exclusion criteria employed by the treatment center are (1) current psychosis; (2) substance dependence; (3) a body-mass-index < 17 (because of the sports modules); (4) antisocial personality disorder; (5) war veterans; (6) clients with a recent severe suicide attempt in the last two months. Clients capable of comprehending the assessments are eligible to participate in the study.

### 2.5 DBT-PTSD

This inpatient, modular DBT-PTSD treatment program of 12 weeks largely follows the original protocol developed by Bohus and colleagues (28, 29). **Table 1** provides an overview of all treatment modalities. Protocol modifications include the addition of four exposure sessions (total 16) and systemic therapy. In general, the treatment consists of three phases and has both fixed and optional modules. In phase 1 (weeks 1 - 2) clients identify values they find important in life and goals they wish to work on during and after finishing the treatment. They also learn to recognize their avoidance mechanisms, determine their index trauma and acquire DBT skills. Trauma-focused therapy is the main focus of phase 2 (weeks 3 - 10). The individual psychotherapy consists primarily of PE, but has elements of cognitive therapy as well. Also, therapists can, after consultation with their client and the treatment team, use EMDR when that seems appropriate. In the third phase (weeks 11-12) the objective is to help clients accept trauma-related elements of their personal history and focus on how they wish to pick-up their daily lives after finishing treatment. Enhancing self-acceptance and self-compassion are integrated throughout the treatment program.

**Table 1:** Overview of modules within clinical DBT-PTSD across the three phases of treatment.

Individual		Group	
Trauma-focused therapy^123^ᵐ Exposure *in vivo* ^23^* Mentor meetings^123^ DBT Diary review^123^ Psychomotor therapy^23^* Systemic therapy^123^*ᵐ	Creative therapy^23^ Nightmare training^23^* Mindfulness^123^ Expert by experience session^123^*ᵐ	Skills training^123^ Psycho-education^12^ Self-worth^23^* Interpersonal effectiveness^23^* Creative therapy^123^*	Psychomotor therapy¹²³ Conquering life³ Mindfulness ¹²³ Sports modules¹²³ᵐ Self-defence^¹²³^

Treatment phase = ¹²³; Optional modules = *; Protocol modifications = ᵐ.

### 2.6 Measurement instruments

**Figure 2:**
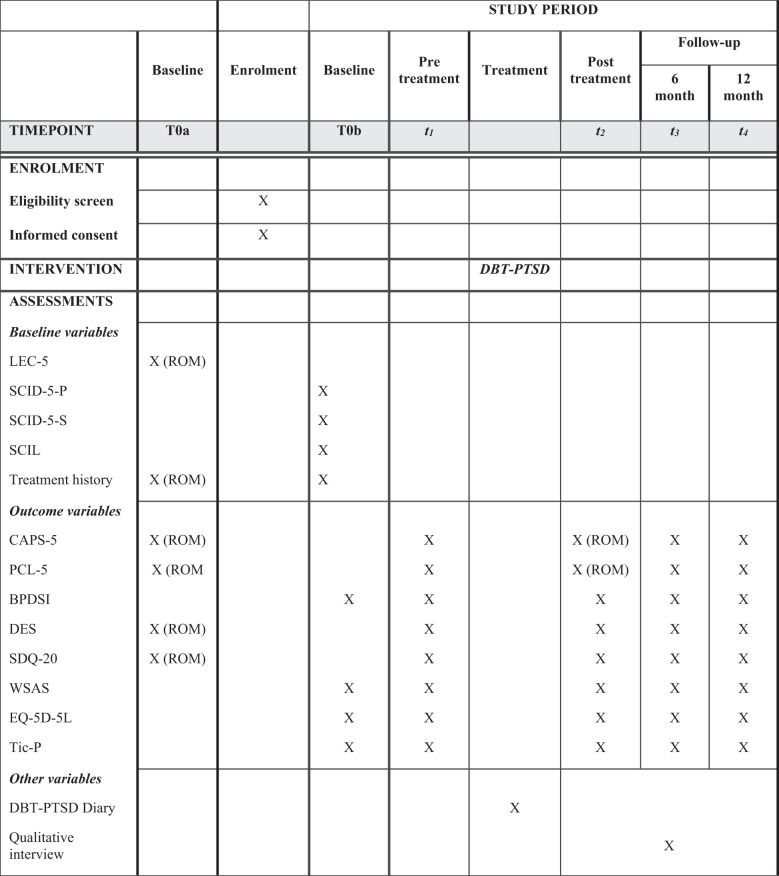
Overview of enrolment, intervention and assessments (based on the spirit guidelines, 36).

An overview of all measurements is provided in **Figure 2** and shows which measures are part of ROM at the treatment center. Treatment history is assessed at baseline. At follow-up, we register treatments that clients received after inpatient DBT-PTSD.

#### 2.6.1 Primary outcome: PTSD

The Life Events Checklist-5 (LEC-5) (37, 38) is a self-report measure that assesses type of trauma. It consists of 16 traumatic events that may lead to PTSD. The LEC-5 is administered a as part of regular diagnostics.

With the Clinician Administered PTSD Scale for DSM-5 (CAPS-5) (39, 40) we measure the presence and severity of PTSD. The CAPS-5 has a range of 0-80 with higher scores indicating more severe PTSD symptoms. In the last week of treatment the week-version of the CAPS-5 is standardly used at the treatment Centre to assess PTSD posttreatment (T2). The CAPS-5 has high interrater reliability (ICC = .98) and internal consistency (α = .90) for measuring PTSD severity. Based on considerations expressed by Larsen et al. (41) and Varker et al. (42), we define treatment response as a decrease of ≥9 points (11%) on the CAPS-5, remission as a CAPS-5 score of <12 and sustained PTSD remission at 6 and 12 months follow-up.

We use the PTSD Checklist for DSM-5 (PCL-5) (43) to assess the self-reported severity of PTSD. This questionnaire consists of 20 items with a 5-point Likert Scale (0-4) and has a range of 0-80. It is sensitive to measure change in PTSD symptoms and has good psychometric properties (43, 44).

#### 2.6.2 Dissociation

Dissociative experiences are assessed with the Dissociative Experience Scale (DES) (45), a self-report questionnaire that consists of 28 questions (range of 0-100). Items are scored on a Likert-scale varying from 0% (never) till 100% (all the time). Reliability and validity have been confirmed (45, 46).

The Somatoform Dissociation Questionnaire-20 (SDQ-20) (47, 48) is a self-report questionnaire that assesses somatoform dissociative symptoms. Two studies demonstrated convergent and criterion-related validity and high internal consistency in Dutch samples (47). Third, we assess the dissociative subtype (depersonalization and derealization) with two items (range of 0-8) of the CAPS-5.

#### 2.6.3 Borderline and parasuicidal behavior

We use the Borderline Personality Disorder Severity Interview (BPDSI) (49) to determine the severity of borderline personality disorder during the past three months. The BPDSI is a 70 item (range 0-90) interview. It has excellent interrater reliability, good internal consistency (α = 0.85) and excellent concurrent and construct validity (49). We use the subscale ‘Parasuicidal’ to assess non-suicidal self-injury and suicidality, because it has good internal consistency (α = .81) (49).

#### 2.6.4 Comorbid disorders

Comorbid personality disorders are assessed with the Structured Clinical Interview for DSM-5 Personality Disorders (SCID-5-P) (50). The SCID-5-P is a semi-structured DSM-5-based interview which assesses the presence of personality disorders. It is supplemented with a screenings questionnaire to shorten the duration of the interview. Interrater reliability of the former version was excellent (51).

Comorbid syndrome disorders are assessed with the Structured Clinical Interview for DSM-5 Syndrome Disorders (SCID-5-S) (52). This semi structured interview aims to assess DSM-5 syndrome disorders. Two studies confirmed the psychometric properties (53, 54).

#### 2.6.5 Social functioning, quality of life and economic evaluation

To study the effects of DBT-PTSD on social functioning, we use the Work and Social Adjustment Scale (WSAS). The WSAS measures impaired functioning due to a disorder and contains five items/domains (work, home management, social leisure activities, private leisure activities and maintaining close relationships). Reliability and validity were demonstrated (56). We operationalize functional recovery with a score of ≤10 on the Work and Social Adjustment Scale.

We assess health-related quality of life with the EQ-5D-5L (55), a self-report questionnaire with excellent psychometric properties (57). It consists of five dimensions: mobility, self-care, usual activities, pain/discomfort and anxiety/depression. Each dimension has five levels: no problems, slight problems, moderate problems, severe problems and extreme problems. We will calculate Quality Adjusted Life Years (Qalys) with the EQ-5D-5L using the Dutch tariff of Versteegh et al. (58).

The TiC-P is a reliable self-report questionnaire that assesses healthcare consumption and productivity loss for clients with a psychiatric disorder (59). The TiC-P has a recall period of three months and aims to facilitate estimation of direct medical costs and productivity costs in paid and unpaid work. It is widely used in the Netherlands for economic evaluations in mental health. The TiC-P captures for instance duration, intensity and type of (follow-up) mental health and other treatment in the waiting period, during the intervention and after discharge, as well as (emergency) admissions to the hospital, before and after discharge.

#### 2.6.6 Client perspectives and individual symptom trajectories

With a qualitative semi-structured interview, we study clients’ experiences with DBT-PTSD. With open questions clients are asked about subjects like, changes they experienced during and after treatment, factors they perceived as helping or hindering the treatment process and their suggestions for improvement. Interviews will take 60 to 90 minutes and will be audiotaped and afterwards verbatim transcribed.

We use the DBT-PTSD diary to assess symptom trajectories and the individual differences herein between clients. The DBT diary was developed and provided by Bohus and colleagues in the Central Institute for Mental Health in Mannheim, Germany. As part of treatment, clients keep a digital daily diary during the twelve weeks of treatment. Items in this diary include among others, PTSD intrusions, sleep, suicidality, problem behavior, happiness, time spent on therapeutic assignments and frequency of exposure assignments. Items vary from nominal, (yes/no), ordinal (5-point Likert scale) to continuous.

#### 2.6.7 Baseline screening

We use the Screener for Intelligence and Learning disabilities (SCIL), which is a valid and reliable screening questionnaire developed to detect an intelligence quotient of ≤ 84, indicated by a cut-off score of 19 or lower (60). Out of consideration for clients’ wellbeing and the validity of the results, clients are offered to stop the interviews or complete only part of the measurements when they have a SCIL score <20, show difficulty understanding the measurements, or seem too distressed.

### 2.7 Data management

Two independent research nurses coordinate data collection. They are the only ones that have access to client information in combination to the data. In a separate registration database (a password protected Excel file), basic personal data like name, sex, date of birth, and where applicable, reasons for non-participation or lost to follow-up of potential participants (i.e. all clients referred to the trauma center for intake that are interested in participation) is stored. The registration database supports the planning of all assessments and is stored on a separate account accessible only to the research nurses. The registration database includes the key to the anonymized data. For the analyses, an anonymized dataset is extracted from the online questionnaire system RoQua (Routine Outcome and Quality Assessment). Data collected specifically for the present study that cannot be stored in the RoQua system, is stored on separate, anonymized (SPSS, Word and MPEG-4 Audio) files. This concerns the treatment history questionnaire and the qualitative interviews. During data collection and analysis, data files are only accessible for the research nurses during data collection. After terminating data collection, the researchers involved in the project can access the anonymized data file. The only exception are the qualitative interviews, which the interviewing researchers (PV and AK) can access during the study.

The datasets of this study will not be publicly available because they contain personal information. Access to the dataset is restricted to project team members. The processed and pseudonymized data can be obtained from the principal investigator upon reasonable request, provided the research question aligns with the informed consent. Study results will be published in international peer-reviewed journals and presented at conferences, regardless of the outcomes.

### 2.8 Sample size calculation

Seven paired-samples t-tests are used to assess short term within-subjects differences in 1. PTSD severity (measured with the CAPS-5 and PCL-5 scores); 2. BPD severity (measured with the BPDSI total score and sum score of subscale Parasuicidal of the BPDSI) and 3. dissociation (measured with the SDQ-20, DES and sum scores of items 19 and 20 of the CAPS-5). To correct for chance capitalization as a result of multiple testing we use a conservative alpha level of 0.01, thereby lowering the chance of a type 1 error. We choose a power of.80. The minimum needed sample size, based to detect a medium difference (of 0.5 SDs) between the two paired scores is N=51 (using G*power) with α=.01 and a power of.80. We anticipate a minimal dropout of about 10%. This is based on the smaller drop-out rates in the RCT of Bohus et al. (2013), in combination with the naturalistic design of the current study which may involve more drop-out. We compensate for possible missing values or loss to follow-up by including an extra 10%, yielding a total sample size of 56 clients.

## 3 Results

### 3.1 Quantitative analyses

Data-analyses will be carried out by using the latest version of R and Rstudio. Analyses will be done on an intention-to-treat basis. Frequencies and percentages will be given for gender, co-morbid disorders, trauma type (sexual/physical abuse <12 years, sexual/physical abuse during adolescence (>12 - <18), sexual/physical abuse adulthood, other trauma), treatment history (types of previous treatment) and drop-out. Means, standard deviations, range and medians will be calculated for age, number of different traumata experienced in life and treatment history in years. To test for selection bias, independent two-sided t-tests will be done comparing the data of the study sample with regular ROM measurements (CAPS-5, PCL-5, SDQ-20 and DES) of non-participants at T0a. For this purpose, a total sample of N=128 (N=64 in each group) is needed with a power of.80 and an alpha of.05.

#### 3.1.1 Short and long term effects of DBT-PTSD

To study short-term within-subjects differences, change scores will be calculated with the first three time points (T2-T1=Δtreatment and T1- T0=Δwaiting-list), resulting in four Δ-variables for PTSD, six for dissociation, two for NNSI and suicidal ideation and two for borderline symptomatology. Two-sided paired-samples t-tests will be conducted to compare the Δtreatment and Δwaiting-list scores. We use two-sided tests to maintain a conservative α level. When the anticipated waiting list period turns out to be less than three months, we will consider excluding the participant from the paired t-test analyses. When, at the end of the study, the waiting list period appears to vary much more than expected, and we might need to exclude too many patients, we will consider alternative statistical analyses that are able to deal with this variability, such as linear mixed model analysis. *Post-hoc* analyses to assess long term treatment effects in PTSD, dissociation, NNSI and suicidal ideation, and borderline symptomatology will be carried out with linear mixed model analyses with time as random effects. We will include two predictors of treatment effects on PTSD severity, as measured with the CAPS-5 and PCL-5 into the PTSD model: 1) baseline severity of BPD symptoms (using the total score on the BPDSI) and 2) cluster C personality disorders (with the sum of the dimensional SCID-5-P scores of the three cluster C personality disorders). Further, we will use linear mixed model analyses to study the effects of DBT-PTSD on social functioning and quality of life over time.

#### 3.1.2 Symptom trajectories with diary data

Finally, as more explorative objectives, we intend to study symptom trajectories on a group and individual level with the diary data. We will use linear mixed model analyses and graphs to show symptom trajectories over time with PTSD intrusive symptoms, emotion dysregulation behaviors (non-suicidal self-injury, high risk behavior, binge eating or self-induced vomiting, alcohol use, drugs/medication use, anger, other problem behavior), suicidality, hours of sleep, happiness and undertaking pleasant activities. Linear mixed model analyses are capable of handling missing data, even when a substantial part of the data is missing. We hypothesize that when PTSD symptoms decrease, improvements on the other variables will follow. To test this hypothesis, we will include PTSD symptoms as a time-varying covariate with a two week lag in our linear mixed models.

Second, we study the degree to which clients invest in their treatment, operationalized by the DBT-PTSD diary items: ‘time spent on therapeutic assignments’ (in minutes) and ‘exposure assignments carried out’ (yes/no), as predictors of treatment outcome. We will use the CAPS-5 and PTSD symptoms of the diary to measure change in PTSD symptoms and the diaries emotion dysregulation behavior items for emotion dysregulation. We hypothesize that clients who are motivated and invest in their treatment, by doing more exposure assignments and spending more time on therapeutic assignments, will benefit more from DBT-PTSD. To test this hypothesis, we will split the sample in half to compare clients with the highest and lowest scores on homework and exposure assignments during the first four weeks and over the whole course of treatment. We will again use linear mixed model analyses with a time x group interaction in our model.

### 3.2 Handling missing data

To thoroughly analyze missing data we will use a combination of graphic inspection with missing data matrix plots and bar charts, calculate frequencies and percentages of missing data for each variable at every time point and use the Little’s test (61). This will enable us to determine the extent of the missingness in our dataset and its nature (missing completely at random or more systematic). In our estimation of possible patterns we will take all the available information into account, like the detailed records that are kept of individual participants during data collection, the COVID-19 pandemic, symptom severity, etc. We intend to use multiple imputation, if this is indeed the most optimal method to handle missing data in our study. Multiple imputation is statistically the most accurate way of dealing with missing data (62). The results of the analyses in 20 imputed datasets will be pooled. We will only delete cases from the analyses when there is too much data (≥50%) missing for multiple imputation. For the linear mixed model analyses we will use the original, unimputed dataset, because these statistical analyses can deal with missing values. The information about missing data in the study will be reported in detail, using the Strengthening the Reporting of Observational Studies in Epidemiology (STROBE) statement for observational studies (63).

### 3.3 Economic evaluation of DBT-PTSD

We will perform a cost-effectiveness and cost-utility analysis from a societal perspective. We calculate healthcare costs and productivity loss over the past three months (measured with the Tic-P), using standard unit cost units as described in the Zorginstituut Nederland (ZiN) guidelines (64). Outcomes consist of PTSD severity (measured with the CAPS-5) and quality-adjusted life-years (QALY’s), which will result in two Incremental Cost-Effectiveness Ratios (ICERs): additional costs for one point improvement in PTSD severity and additional costs per Qaly. Parallel to the analysis of effectiveness, in the cost-effectiveness analysis we also compare within-subjects: intervention versus waiting list. To obtain an estimate on longer-term cost-effectiveness, costs and effects of inpatient DBT-PTSD will be extrapolated from the six and twelve month follow-up data to a five year outcome, using four different scenario’s to gain insight into the upper and lower limits of the actual cost-effectiveness: 1) Care consumption and PTSD severity will remain what they were during the last six months follow-up; 2) Care consumption and PTSD severity are (back) at baseline levels immediately after the end of 1 year follow-up (pessimistic); 3) Care consumption and PTSD severity will gradually decrease after the follow-up has ended, with 25% in total over five years (optimistic); 4) Care consumption and PTSD severity will gradually increase again (after initial reduction) after the follow-up ends, until the baseline levels are reached, linear in five years (moderately pessimistic). Bootstrapping and cost-effectiveness acceptability curves (CEAC) will be used to quantify the uncertainty around the ICERs. We will use the CHEERS 2022 statement in reporting our outcomes (65).

### 3.4 Qualitative analysis

A qualitative study will be conducted to better understand clients’ (short- and long-term) experiences with inpatient DBT-PTSD. We include clients in this part of the study, based on maximum variation sampling (age, gender, region of residence, treatment year) and expect to reach data saturation with approximately 15 participants. We will carry out a phenomenological reflective life world approach as described by van der Meide (2014) (66). Interviews are audiotaped and later verbatim transcribed. The first four interviews are transcribed by a researcher (PV) and the remaining by two independent (reintegrating) colleagues of the mental health facility. As it is an iterative process, analysis starts immediately after the first round of interviews. The supervising researcher (MA), specialized in qualitative interviews, and the two interviewing researchers (AK and PV) will be involved in the analyses. We follow the four phases of the reflective life world approach as described by Van der Meide (2014). The first phase consists of re-reading and exploring the interview data. Secondly, we will identify different units of meaning (coding). The supervisor and the researcher will code the first three interviews independently. An inductive coding approach will be used to categorize the variety of clients’ experiences. Throughout the data collection and analysis, codes and clusters are open for re-examination to get closer to the concepts and experiences targeted by this study. After the first round of manual coding, transcripts will be coded further using Atlas.ti 24 (67). Third, we will cluster the meaningful units that are related. As a fourth step, we will formulate the essence of the phenomenon that is being studied. To enhance the quality of the study, the analysis of the first six interviews will be discussed in a supervision group of qualitative researchers. Additionally, peer-debriefing with an affiliated researcher (MA) will be utilized to overcome potential inaccurate assumptions or biases. We will use the COREQ guidelines throughout the process of reporting (68).

## 4 Discussion

This paper describes the design of a naturalistic study aimed at examining short and long term effects of inpatient DBT-PTSD. It will provide information about the effects on PTSD, dissociation, parasuicidal behavior, borderline symptomatology, social functioning, health-related quality of life and cost-effectiveness. It is the first study that includes both the severity of BPD and cluster C personality disorders as predictors of treatment effects. Another novel aspect is the qualitative study of clients experiences with DBT-PTSD. Finally, the use of diary data will show individual symptom trajectories and whether clients’ investment into their treatment in terms of homework predicts their treatment results.

The first studies on DBT combined with trauma-focused treatment, with varying designs and sample sizes, have shown promising results in clients with severe PTSD, emotion regulation problems and comorbid BPD (30). First, pilot studies and a naturalistic study on outpatient DBT with PE indicate that PTSD symptoms, suicidality, self-injurious behavior and social functioning improve in women with PTSD and BPD (69–71). In an RCT, outpatient Cognitive Processing Therapy and outpatient DBT-PTSD were both effective in reducing PTSD symptoms in women with a history of childhood abuse (72). DBT-PTSD led to greater remission and recovery rates and less drop-out compared to Cognitive Processing Therapy (respectively 25.5% vs. 39.0%). The inpatient variant of DBT-PTSD has a shorter, intensive treatment program. Compared to a waiting list group in which TAU was continued DBT-PTSD was effective in reducing PTSD symptoms (29). In an observational study DBT-PTSD outperformed an inpatient treatment that included trauma-focused treatment on PTSD, dissociation and disturbances in self-organization (32).

Replication of these results in mental healthcare settings beyond those of the original developers is essential to inform treatment guidelines. Also, information about treatment predictors (beside BPD), symptom trajectories, cost-effectiveness and clients’ experiences with DBT-PTSD is currently limited or absent. The present study addresses these knowledge gaps and extends the evidence base for a broader client group (both men and women) with severe PTSD and comorbid (personality) symptoms with a more varied trauma history. Adjacent to this, we intend to increase knowledge of both borderline and cluster C personality disorder symptoms as predictors of inpatient DBT-PTSD outcomes. Comorbid personality disorders negatively impact PTSD treatment outcomes compared to outcomes of clients without comorbid personality disorders, as was shown in a recent meta-analysis (24). On the other hand, comorbid BPD was not predictive of treatment outcome in an RCT on inpatient DBT-PTSD (29). As an additional objective, next to symptomatic improvement, we study the short and long term treatment effects on social functioning and quality of life. The results of Bosch et al. (2020), who studied the effect of Cognitive Processing Therapy in PTSD clients with early trauma, suggests that symptom reduction does not automatically lead to improvement of quality of life. Further, to our knowledge, our study is the first to gain insight into symptom trajectories and clients’ experiences with DBT-PTSD, by using daily diaries and qualitative interviews. Finally, as a 12-week inpatient treatment is costly, it seems highly relevant to study its incremental cost-effectiveness on PTSD severity and cost-utility in terms of QALYs, compared to outpatient TAU.

### 4.1 Strengths and limitations

Ideally, we would have conducted an RCT and compare DBT-PTSD with an alternative evidence based PTSD treatment, as this is considered the golden standard. However, a pilot with ten clients showed that once referred, all refused randomization to either inpatient DBT-PTSD or an intensive inpatient treatment of eight days at another location in the Netherlands. A preference trial turned out not feasible either. Apparently, once referred to a treatment program, clients are focused on and prepared by their referrers to start with this particular treatment. Furthermore, as clients are referred to DBT-PTSD by a large number of organizations and general practitioners located throughout the Netherlands, it was not possible to randomize at an earlier stage. Finally, comparing DBT-PTSD to a waiting list control group was considered unethical by the local medical ethical committee, because one group of clients would have waited longer for treatment.

The lack of control for possible confounding variables is of course a limitation. However, by including measurements before and after the waiting list period, clients may serve as their own control. Moreover, most clients referred to inpatient DBT-PTSD have a history of (severe) childhood traumatization and suffered from PTSD and other symptoms for a long period of time. Hence, it seems unlikely that their symptoms would significantly improve during a waiting list period. On the other hand, clients are free to continue or start any other treatment or counselling during the waiting list period. In our naturalistic study design we cannot rule out TAU treatment effects in our waiting list control period. Another limitation is that we cannot be certain that other factors than DBT-PTSD influence the six and twelve-months follow-up measurements of symptoms, social functioning and quality of life. To gain some insight in this, we gather data about treatments between posttreatment and the follow-up measurements. A final limitation to consider is that the CAPS-5 measures PTSD related to one index trauma. Clients referred to the DBP-PTSD program have often experienced multiple traumas. During the repeated measures with the CAPS-5, the interviewers will refer to the index trauma at T0. This may bias the results in overestimating treatment success. However, besides the CAPS, we use the PCL-5 and the DBT-PTSD diary and both measure PTSD symptoms irrespective of trauma experiences.

Our within-subject observational design has several advantages. First, it allows us to study the short and long-term effects and individual treatment trajectories of clients with severe PTSD and co-morbid symptoms, including those with non-suicidal self-injury and suicidal thoughts, in a naturalistic setting. Second, all clients that are considered eligible for DBT-PTSD by de staff of the treatment center can participate in the study. The absence of exclusion criteria enlarges the generalizability of the results. Third, it is a pragmatic study, in the sense that clinicians and clients are free to design a personal treatment plan in which they choose optional modules they consider helpful in achieving clients’ personal goals. This way, study results reflect DBT-PTSD treatment as it is given in everyday clinical practice. Furthermore, the study is carried out by researchers who were not involved in the design of the treatment program, which enhances the objective evaluation and generalizability of DBT-PTSD. Finally, the qualitative study may lead to important insights into client perspectives on treatment effects and helpful ingredients of DBT-PTSD.

### 4.2 Conclusion

In conclusion, the Trauma Therapy Study aims to extend the evidence base of inpatient DBT-PTSD for clients with severe PTSD and co-morbid psychiatric symptoms as a consequence of a traumatic history in a day-to-day healthcare setting. Beside extending nomothetic knowledge, (group level knowledge), idiographic knowledge is gained by including qualitative interviews and diary data. Perspectives of clients who participated in DBT-PTSD can inspire improvements to the treatment program. Insight into individual symptom trajectories may inform clinicians to adjust future treatments more timely. Growing evidence of effective treatments for this client group and knowledge of prognostic factors, gives both clients and involved clinicians the opportunity to choose the most appropriate treatment through shared decision-making (73).
